# MUTYH mediates the toxicity of combined DNA 6-thioguanine and UVA radiation

**DOI:** 10.18632/oncotarget.3037

**Published:** 2014-12-02

**Authors:** Francesca Grasso, Vitalba Ruggieri, Gabriele De Luca, Paola Leopardi, Maria Teresa Mancuso, Ida Casorelli, Pietro Pichierri, Peter Karran, Margherita Bignami

**Affiliations:** ^1^ Department of Environment and Primary Prevention, Istituto Superiore di Sanità, Rome, Italy; ^2^ Department of Science, University Roma Tre, Rome, Italy; ^3^ Laboratory of Pre-Clinical and Translational Research, IRCCS, Referral Cancer Center of Basilicata, Rionero in Vulture, Italy; ^4^ Laboratory of Radiation Biology and Biomedicine, Agenzia Nazionale per le Nuove Tecnologie, l'Energia e lo Sviluppo Economico Sostenibile (ENEA) CR-Casaccia, Rome, Italy; ^5^ Department of Immunohematology and Transfusion Unit, Azienda Ospedaliera Sant'Andrea, Rome, Italy; ^6^ Cancer Research UK London Research Institute, Clare Hall Laboratories, South Mimms, Herts, UK

**Keywords:** MUTYH, 6-thioguanine, azathioprine, UVA

## Abstract

The therapeutic thiopurines, including the immunosuppressant azathioprine (Aza) cause the accumulation of the UVA photosensitizer 6-thioguanine (6-TG) in the DNA of the patients' cells. DNA 6-TG and UVA are synergistically cytotoxic and their interaction causes oxidative damage. The MUTYH DNA glycosylase participates in the base excision repair of oxidized DNA bases. Using *Mutyh*-nullmouse fibroblasts (MEFs) we examined whether MUTYH provides protection against the lethal effects of combined DNA 6-TG/UVA. Surprisingly, *Mutyh*-null MEFs were more resistant than wild-type MEFs, despite accumulating higher levels of DNA 8-oxo-7,8-dihydroguanine (8-oxoG). Their enhanced 6-TG/UVA resistance reflected the absence of the MUTYH protein and MEFs expressing enzymatically-dead human variants were as sensitive as wild-type cells. Consistent with their enhanced resistance, *Mutyh*-null cells sustained fewer DNA strand breaks and lower levels of chromosomal damage after 6-TG/UVA. Although 6-TG/UVA treatment caused early checkpoint activation irrespective of the MUTYH status, M*utyh*-null cells failed to arrest in S-phase at late time points. MUTYH-dependent toxicity was also apparent *in vivo*. *Mutyh^−/−^*mice survived better than wild-type during a 12-month chronicexposure to Aza/UVA treatments that significantly increased levels of skin DNA 8-oxoG. Two squamous cell skin carcinomas arose in Aza/UVA treated *Mutyh^−/−^* mice whereas similarly treated wild-type animals remained tumor-free.

## INTRODUCTION

Reactive oxygen species (ROS) are generated in living cells as by-products of incomplete mitochondrial electron transfer, during the inflammatory response and following exposure to radiation or chemicals. ROS production in excess of the cellular antioxidant capacity results in a state of oxidative stress in which DNA is vulnerable to damage. One of the most abundant ROS-induced DNA lesions, 8-oxo-7,8-dihydroguanine (8-oxoG) is mutagenic because it mispairs with adenine during DNA replication. 8-oxoG-induced G:C to T:A transversions [1] are prevented by base excision repair (BER) involving the concerted action of the MUTYH and OGG-1 DNA glycosylases [for reviews see refs. 2-4].

Following MUTYH-dependent removal of adenine from 8-oxoG:A mispairs and excision of the abasic site, the gap is filled by DNA polymerase λ which preferentially incorporates dCMP opposite the persisting 8-oxoG [5]. Faithful repair is then completed by DNA ligase and flap endonuclease 1 *via* the long-patch BER pathway [6]. Normal base pairing is restored by subsequent OGG1-mediated BER which removes 8-oxoG from the resulting 8-oxoG:C base pairs. Mismatch repair (MMR), a major replication error-correcting pathway [7], can also prevent mutations arising at mismatches containing oxidized bases [8,9].

The importance of protection against ROS-induced DNA damage is illustrated by the association between *MUTYH* gene mutations and MUTYH-Associated Polyposis (MAP), a heritable syndrome linked to an increased colorectal cancer risk [10,11]. Biallelic *MUTYH* mutations confer a spontaneous mutator phenotype in human cell lines [12,13] and in mice [14-16]. Consistent with a role in BER, *MUTYH*-defective cell lines are sensitive to oxidants (H_2_O_2_, KBrO_3_, t-butyl hydroperoxide) [16-18].

6-thioguanine (6-TG) is incorporated into the DNA of patients undergoing treatment with thiopurines including azathioprine (Aza). High levels of DNA 6-TG are cytotoxic, probably due to aberrant processing of 6-TG-containing base pairs by MMR [for a review see 19]. In addition to its direct toxicity, subtoxic levels of DNA 6-TG interact with UVA to generate ROS. These cause multiple forms of potentially lethal DNA damage [20-24], including DNA breakage in S phase [20,21,24]. In view of the acknowledged role of MUTYH at replication [25,16], we investigated whether it protects against the cytotoxicity of combined 6-TG/UVA. Unexpectedly, cells derived from *Mutyh^−/−^* mice were resistant to 6-TG/UVA. In addition, *Mutyh^−/−^* mice also survived long-term chronic treatment with Aza/UVA better than their wild-type counterparts. Squamous cell carcinomas only developed in *Mutyh^−/−^* mice, however, suggesting that protection against toxicity conferred by a defective *Mutyh* gene does not extend to protection against cancer development.

## RESULTS

### MUTYH loss and resistance to 6-TG/UVA

**Figure 1:**
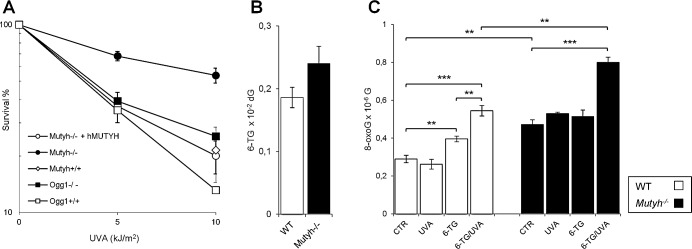
Cytotoxicity and 8-oxoG levels induced by 6-TG/UVA A) Cytotoxicity by combined exposure to 6-TG and UVA in MEFs derived from *Mutyh*^−/−^ (closed circle), *Mutyh*^−/−^ + *hMUTYH* cDNA (open circle), Mutyh^+/+^ (open diamond), *Ogg1*^−/−^ (closed square) and *Ogg1*^+/+^ (open square) mice. Cells were grown for 48h in medium containing 60nM 6-TG, and irradiated with the indicated UVA doses. Clonal survival was measured after 10 days. Results are the mean ± SE of 3 independent measurements. B) DNA 6-TG levels in WT (white bars) and *Mutyh*^−/−^ (black bars) MEFs. DNA 6-TG was measured by HPLC and UV absorption spectrum in cells cultivated for 48h in 6-TG (0.6μM). Results are the mean ± SE of 6-10 independent measurements. C) DNA 8-oxoG levels in WT (white bars) and *Mutyh*^−/−^ (black bars) MEFs. DNA 8-oxoG was measured by HPLC/EC in untreated cells (CTR), cells cultivated for 48h in 6-TG (0.6μM), irradiated with UVA (10kJ/m^2^) or exposed to a combined 6-TG/UVA treatment. Results are the mean ± SE of 3-5 independent measurements. **P≤0.005 ***P≤0.0005 (Student's *t*-test).

We compared the 6-TG/UVA sensitivity of *Mutyh^−/−^* MEFs and the same cells in which the repair defect had been corrected by expressing the nuclear isoform of wild-type human MUTYH [16, 26]. Cells that had been allowed to incorporate 6-TG into DNA by growth for 48h in 6-TG-supplemented medium were UVA irradiated and survival was determined by clonal assay. 6-TG-treated *Mutyh^−/−^* cells were surprisingly resistant to UVA compared to their corrected counterparts (Figure 1A). In contrast, the UVA sensitivity of 6-TG-treated *Ogg1^−/−^* MEFs was similar to that of wild-type cells (*Mutyh^+/+^*, *Ogg1^+/+^* and *Mutyh^−/−^* + *hMUTYH*). (We designate *Mutyh^−/−^* + *hMUTYH* cells as wild-type from here). The low UVA doses alone did not affect survival and 6-TG treatment in the absence of irradiation reduced cloning efficiency by < 20% (data not shown). Since the extent of DNA substitution by 6-TG was similar in *Mutyh^−/−^* and wild-type MEFs (Figure 1B), the resistance to 6-TG/UVA associated with MUTYH loss cannot be ascribed to differential DNA 6-TG accumulation.

MUTYH inactivation is associated with higher steady-state levels of DNA 8-oxoG (0.47 *versus* 0.29 per 10^−6^ dG in *Mutyh^−/−^* and wild-type MEFs, respectively) (Figure 1C). Irradiation with 10 kJ/m^2^ UVA did not alter basal DNA 8-oxoG levels in any of the cell lines. Exposure of wild-type cells to 6-TG increased DNA 8-oxoG levels by 1.4-fold and these were increased further to 1.9-fold by UVA irradiation (Figure 1C). In contrast, growth of *Mutyh^−/−^* MEFs in 6-TG did not measurably increase DNA 8-oxoG levels whereas combined 6-TG/UVA treatment caused a 1.7-fold increase. The number of oxidized purines following 6-TG/UVA exposure is, however, significantly higher in *Mutyh^−/−^* cells than in wild-type cells (0.8 ± 0.03 *versus* 0.54 ± 0.03, respectively; P<0.005, Student's *t*-test) (Figure 1C).

These findings indicate that, as in human cells [20], 6-TG and UVA are synergistically toxic to mouse fibroblasts. They indicate further that, despite reducing the burden of DNA 8-oxoG induced by this treatment, MUTYH actually contributes to its toxicity.

### 6-TG/UVA resistance requires the absence of MUTYH

**Figure 2:**
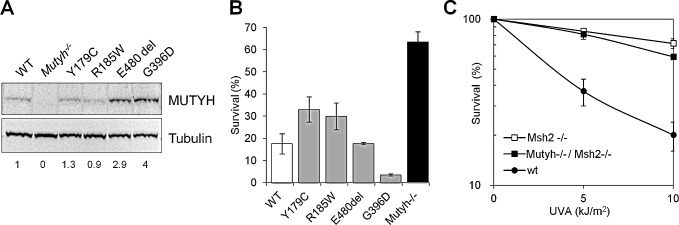
Cytotoxicity of 6-TG/UVA in MEFs expressing MUTYH variants and/or defective in MSH2 A) A representative western blot of the MUTYH protein and β-tubulin used for normalization. Numbers underneath the blot indicate MUTYH expression values normalized on the WT protein. B) Cytotoxicity by combined exposure to 6-TG/UVA in *Mutyh*^−/−^ (black bars), *Mutyh*^−/−^ + *MUTYH* cDNA (WT, open bars), *Mutyh*^−/−^ + variant *MUTYH* cDNAs (grey bars<2). Survival was measured as clonal efficiency 10 days after a 48h growth in 60nM 6-TG and UVA irradiation. Results are the mean ± SE of 2-4 independent measurements. C) Survival was measured as clonal efficiency in WT, *Msh2*^−/−^ and *Mutyh*^−/−^*/Msh2*^−/−^ MEFs after a 48h growth in 60nM 6-TG and UVA irradiation. Values are mean ± SE of 3 independent measurements.

To investigate how MUTYH affects 6-TG/UVA resistance in *Mutyh*^−/−^ MEFs, we examined the responses of a series of *Mutyh*-null cell lines expressing variant forms of *MUTYH*. The p.Y179C, p.R185W and p.G396D missense variants and the p.E480del in-frame deletion were all identified in MAP patients [16]. Each variant is defective in MUTYH DNA glycosylase activity [27] and they are expressed in *Mutyh*^−/−^ cells at levels ranging from 1 to 4-fold that of the corresponding wild-type protein (Figure 2A). None of the MEFs expressing the mutant MUTYH proteins was resistant to 6-TG/UVA. Sensitivity was approximately correlated with the level of MUTYH protein expression (Figure 2B). We conclude that 6-TG/UVA resistance of *Mutyh*-null cells is unrelated to the canonical DNA glycosylase function of MUTYH, but instead reflects the absence of the MUTYH protein.

Since 8-oxoG:A mismatches can also be recognized by the MMR MutSα complex [8,9], a MSH2/MSH6 heterodimer, we examined the response to 6-TG/UVA treatment of *Msh2^−/−^* MEFs. These cells were also resistant to 6-TG/UVA and the extent of their resistance was similar to that of *Mutyh^−/−^* MEFs (Figure 2C). In addition, the sensitivity of doubly deficient *Msh2^−/−^/Mutyh^−/−^* MEFs was indistinguishable from that of the single knockout cells (Figure 2C).

These data indicate that the absence of either the Mutyh or the Msh2 protein confers tolerance to killing induced by 6-TG/UVA. Their effects on sensitivity are epistatic suggesting that the known interaction between MUTYH and MutSα [28] might underlie this tolerance.

### Cell cycle perturbation by 6-TG/UVA

**Figure 3:**
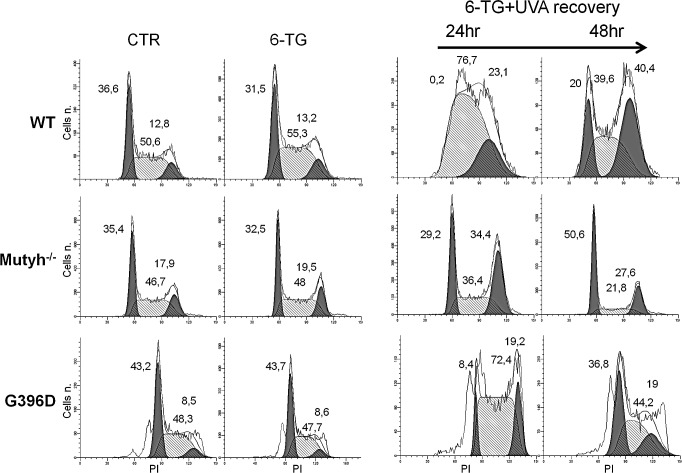
Cell cycle analysis after 6-TG/UVA Cell cycle progression in WT, *Mutyh*^−/−^ and G396D-expressing MEFs. Cells were grown for 24h in 0.6μM 6-TG or 6-TG followed by UVA irradiation and sampled for analyses at the indicated time points. The percentage of cells in G1, S and G2 phases of the cell cycle are also indicated.

The absence of the MUTYH protein had profound effects on cell cycle progression after 6-TG/UVA treatments. In wild-type MEFs, 6-TG/UVA caused a pronounced slowing of progression through the S phase (>75% of the cells were blocked in the S phase at 24h) followed by an accumulation in G2 at 48h (Figure 3). In contrast, the *Mutyh^−/−^* cells did not accumulate in S phase, progressed into the G2-M phase at 24h (>34% of the cells) and in G1 at 48h. The behaviour of G396D-expressing MEFs resembled that of wild-type cells, with a similar increase in S-phase arrest and continuing perturbation at 48h post-treatment. Cell cycle progression was not affected by treatment with 6-TG or UVA alone (Figure 3 and data not shown).

These data suggest that the presence of a MUTYH protein, either active or inactive, affects the progression of 6-TG/UVA treated cells through S phase. The similar cell cycle effects in cells expressing wild-type or mutant MUTYH is consistent with their similar cytotoxic response to 6-TG/UVA.

### Strand breaks, checkpoint activation and chromosomal damage

**Figure 4:**
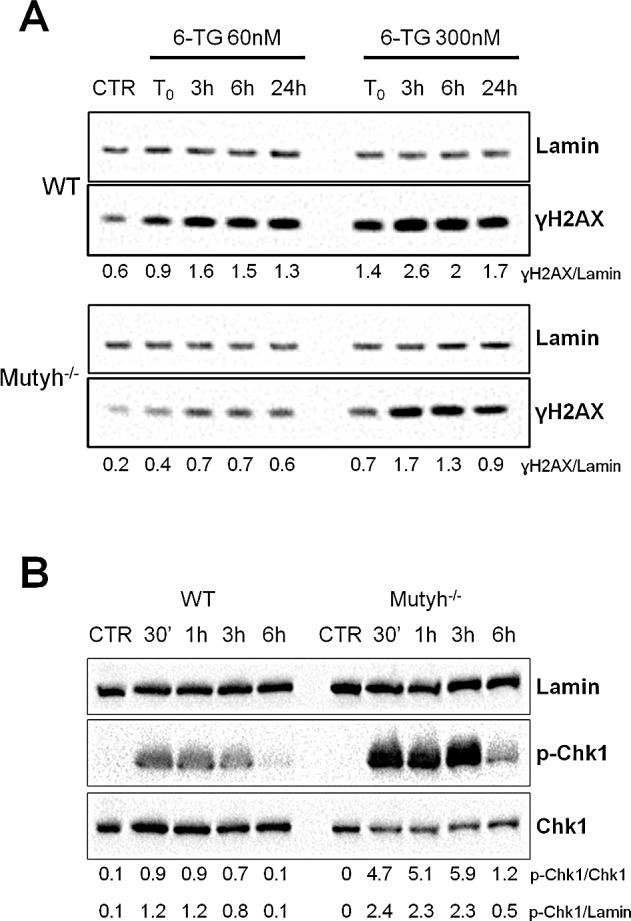
Strand breaks and check-point activation after 6-TG/UVA treatment A) A representative western blotting of ɣH2AX and Lamin proteins in WT and *Mutyh*^−/−^ cell lines at various time points after 6-TG/UVA treatment (48h growth in 60nM or 300nM 6-TG followed by UVA irradiation). Under the blot values of normalized ɣH2AX expression as ɣH2AX/Lamin ratio are shown. B) A representative western blotting of p-Chk1, total Chk1 and Lamin proteins in WT and *Mutyh*^−/−^ MEFs at various time points after 6-TG/UVA treatment (48h growth in 0.6μM 6-TG followed by UVA irradiation). Under the blot values of normalized p-Chk1 expression as p-Chk1/Chk1 or p-Chk1/Lamin ratio are shown.

6-TG/UVA treatment causes DNA single and double strand breaks (DSBs) [24,29]. We investigated whether the presence of MUTYH influenced DNA break formation. Cells grown for 24h in 6-TG (60nM or 300nM) were UVA irradiated and the phosphorylation of histone H2AX (γH2AX) was analysed by western blotting. In wild-type cells, γH2AX was detectable immediately after UVA irradiation, plateaued between 3h and 6h and decreased thereafter (Figure 4A). A similar trend was observed in *Mutyh^−/−^* MEFs, although the extent of H2AX phosphorylation was clearly diminished in comparison to wild-type cells (Figure 4A). We also noticed that the basal level of γH2AX appeared to be lower in the *Mutyh^−/−^* MEFs. A similar analysis of G396D-expressing MEFs treated with the low dose of 6-TG, indicated that γH2AX levels were comparable to those in wild-type cells. At higher 6-TG doses, DNA breaks were more persistent in cells expressing mutant MYH than in wild-type MEFs (Figure S1).

To investigate activation of the S phase checkpoint, the serine 345 phosphorylated form of Chk1 (p-Chk1) was examined at 0.5h, 1h, 3h and 6h post 6-TG/UVA treatment. Chk1 phosphorylation was rapid following treatment of both wild-type and *Mutyh^−/−^* cells (Figure 4B). A significant quantitative difference was apparent between the two genotypes. After comparable treatments, the extent of Chk1 phosphorylation was higher in *Mutyh^−/−^* cells than in wild-type cells.

The prompt activation of Chk1 suggests that checkpoint signalling is correctly initiated in *Mutyh*-defective MEFs, while the reduced H2AX phosphorylation could be related to the absence of a secondary DNA lesion associated with the presence of the MUTYH protein.

**Figure 5:**
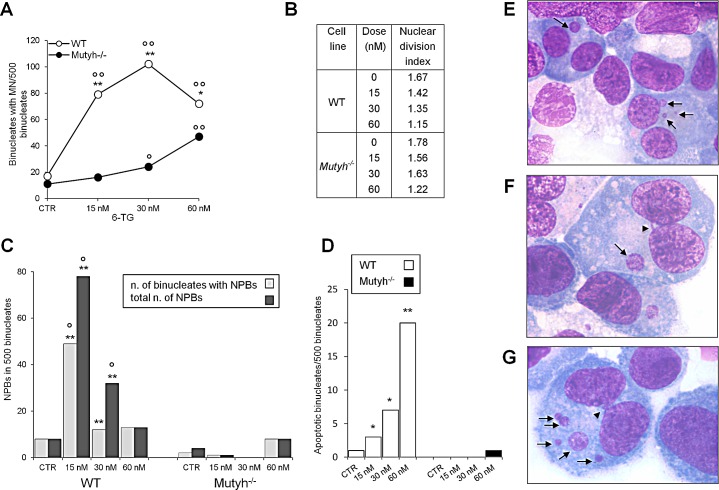
Chromosomal damage after 6-TG/UVA treatment A) Induction of MN by exposure to 6-TG/UVA. Number of binucleated cells with MN in WT (white circles) and *Mutyh*^−/−^ (black circles) MEFs following 6-TG/UVA treatment (48h growth at various doses of 6-TG followed by UVA irradiation). *P<0.05, **P≤0.001 (χ² test: WT *vs Mutyh*^−/−^). °P<0.05; °°P<0.001 (χ² test: treated vs control). B) Nuclear division index relative to WT and *Mutyh*^−/−^ MEFs cultivated in increasing 6-TG concentrations and UVA irradiated. C) Induction of NPBs by exposure to 6-TG/UVA. Number of binucleated cells with NPBs (light gray) and total number of NBPs (dark gray) in WT and *Mutyh*^−/−^ cells MEFs exposed to 6-TG/UVA (experimental conditions as described in A) **P<0.001 (χ² test: WT *vs Mutyh*^−/−^). °P<0.001 (χ² test: treated vs control). D) Number of apoptotic binucleated cells in WT (white bars) and *Mutyh*^−/−^ (black bars) MEFs following 6-TG/UVA (experimental conditions as described in C). *P<0.05, **P≤0.001 (χ² test: WT *vs Mutyh*^−/−^). E) A representative image of MN (indicated by arrows) in the cytoplasm of two binucleated cells. F) A representative image of an NPB connecting two nuclei in the same cell (arrowhead). In the same binucleated cell a MN is also visible (arrow). G) A representative image of an apoptotic binucleated cell containing five MN (arrows) and one NPB (arrowhead). All images are X 1000.

Aza induces chromosomal aberrations [30]. To examine whether MUTYH influences 6-TG/UVA-induced chromosomal damage, we compared micronucleus (MN) formation in 6-TG/UVA treated wild-type and *Mutyh*^−/−^ MEFs (Figure 5A). 6-TG/UVA treatment increased MN frequency in both cells. The effect was, however, much more pronounced in wild-type compared to *Mutyh^−/−^* cells. MN frequencies were significantly increased at 15 nM and maximal at 30nM 6-TG. In *Mutyh^−/−^* cells, the increase in MN frequency was only significant at the higher 6-TG doses (30 and 60 nM) (Figure 5A). Nuclear division indexes confirmed that MN were scored in cell populations showing similar proliferation rates (Figure 5B).

In the same populations of binucleate cells used to determine MN frequencies, we also analysed the formation of nucleoplasmic bridges (NPBs) derived from dicentric chromosomes caused by misrepair of double-strand DNA breaks or telomere end fusions [31]. 6-TG/UVA induced a significant increase in NPBs only in wild-type cells (Figure 5C). Finally, in the same populations of binucleate cells, we observed a dose-dependent increase in the percentage of apoptotic wild-type cells. Apoptosis was almost undetectable in the *Mutyh^−/−^* MEFs (Figure 5D). Representative images of MN, NPBs and apoptotic cells are shown in Figure 5E-F.

These data indicate that whereas 6-TG/UVA toxicity is associated with extensive chromosomal damage in wild-type cells, this damage is barely detectable in the resistant *Mutyh^−/−^* cells.

### ***Mutyh***^−/−^ mice are resistant to the toxicity of combined Aza/UVA treatment but develop skin cancer

**Figure 6:**
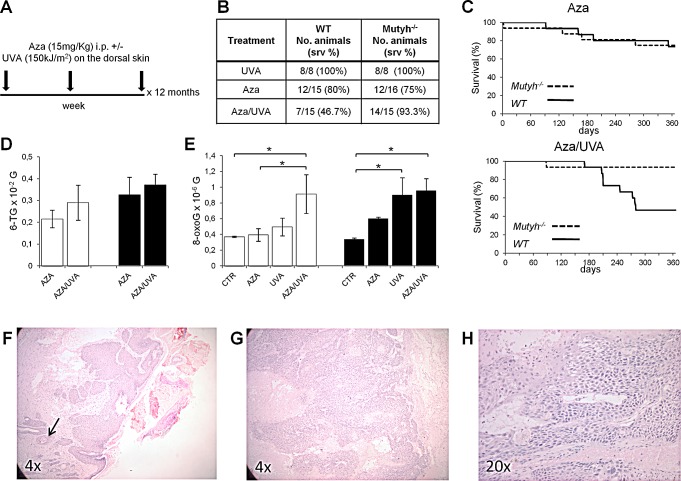
Survival, skin DNA 8-oxoG levels and tumor onset in WT and mice treated with Aza/UVA A) Schematic representation of the treatment. B) Summary of survival data of WT and *Mutyh*^−/−^ mice treated with Aza, UVA or the combined treatment as indicated in A). C) Kaplan–Meier curves of Aza and Aza/UVA treated WT (solid line) and *Mutyh*^−/−^ (dotted line) mice. D) DNA 6-TG levels in the skin of WT (white bars) and *Mutyh*^−/−^ (black bars) mice following Aza or Aza/UVA combined treatment. Data are mean ± SE of 11-19 animals/genotype. E) DNA 8-oxoG levels in the skin of WT (white bars) and *Mutyh*^−/−^ (black bars) mice following Aza, UVA or combined treatment. Data are compared to historical controls (CTR) and they are mean ± SE of 16-25 animals/genotype. *P≤0.05 (Student's t-test). F - H) Representative histological images of SCCs from two Aza/UVA treated *Mutyh*^−/−^ mice. F) Well-differentiated Grade I SCC showing full thickness epidermal atypia and involvement of hair follicles. Keratinization-induced “pearl-like” structures (dermal nests of keratinocytes attempting to mature in a layered fashion containing keratinizing cells and horny pearls) are indicated by an arrow. G) Poorly-differentiated Grade III/IV SCC. H) A higher magnification of the tumor shown in G).

To analyse the effects of 6-TG/UVA *in vivo*, wild-type and *Mutyh*-defective C57BL6 mice were divided in three groups: UVA alone (Group I, 8 animals/genotype), Aza alone (Group II, 15 and 16 wild-type and *Mutyh^−/−^* mice, respectively) and Aza/UVA (Group III, 15 animals/genotype). Group II mice were given 15 mg/kg Aza i.p. All irradiated animals received 150 kJ/m^2^ UVA on shaved dorsal skin. Group III mice were irradiated 1 h after each Aza injection (Figure 6A). All procedures were repeated three times/week for 12 months. Immunosuppression in Aza-treated animals was verified by mixed lymphocyte reaction after 4-weeks treatment (data not shown). As previously reported (32) repeated dosage with 15 mg/kg Aza was well-tolerated and caused only low-level toxicity in both *Mutyh^−/−^* and wild-type animals (Figure 6B and C). In contrast, in Groups III (Aza/UVA) we observed a significant difference in survival between *Mutyh^−/−^* and wild-type mice (p=0.019 by Log-rank test) (Figure 6B and D). At 12 months, 14/15 (>90%) *Mutyh*-defective animals were still alive compared to 7/15 (46.7%) wild-type mice. As expected (33), UVA alone did not cause any discernable skin damage (sunburn cells) in either genotype and 12-month survival was 100% in both Groups I (Figure 6B).

When 6-TG was measured in skin DNA at the end of the treatment (12 months), similar levels of substitutions were found in animals of both genotypes (Figure 6D). DNA 8-oxoG levels were also measured in animals of the three groups. In comparison to historical controls, there was a significant increase of DNA 8-oxoG in Aza/UVA-treated wild-type animals (p=0.02, Student's *t* test) (Figure 6E). In *Mutyh^−/−^* mice a trend of increased DNA oxidation was observed in all conditions reaching statistical significance in UVA- and Aza/UVA-treated animals (Student's *t* test: p=0.06, p=0.03 and p=0.02 in Aza-, UVA- and Aza/UVA treatments, respectively) (Figure 6E). A comparison between Group III wild-type and *Mutyh^−/−^* mice shows that DNA 8-oxoG levels did not differ between the two genotypes (Figure 6E). Similar analyses in spleen and liver samples did not identify any increase in DNA 8-oxoG levels in any of the three groups in these internal organs (data not shown). Thus, as expected the skin is the preferential target for DNA oxidation by Aza/UVA in both wild-type and *Mutyh^−/−^* animals. The skin DNA of Mutyh knockout animals is also susceptible to oxidation by 6-TG and UVA individually.

When histopathological examinations were performed on the skin of surviving animals, two tumors were identified in two *Mutyh^−/−^* mice exposed to Aza/UVA (Figure 6F-H). One was a well-differentiated Grade I squamous cell carcinoma (SCC), showing full-thickness epidermal atypia and the involvement of hair follicles (Figure 6F). The second was a poorly-differentiated SCC (Grade III/IV), with a more advanced anaplastic appearance (Figure 6G). This tumor displays a combination of conventional SCC cells and bundles of spindle shaped cells with elongated nuclei (Figure 6H).

Taken together, these *in vivo* experiments demonstrate that the involvement of the Mutyh protein in mediating the response to damage induced by combined Aza/UVA extends to the intact animal. They are also consistent with the possibility that the absence of Mutyh increases the susceptibility to carcinogenesis induced by photoactivation of DNA 6-TG.

## DISCUSSION

SCC occurs up to 250 times more frequently in organ transplant patients, most of whom will have been immunosuppressed with Aza, than in the general population [34]. Aza is a photosensitizer and sunlight exposure is an acknowledged co-factor in the increased cancer risk [for reviews see [19,35]. Indeed DNA 6-TG, the ultimate metabolite of Aza, interacts with UVA to generate ROS that cause extensive DNA damage including the DNA 8-oxoG that we confirmed in this study [20-24].

The major novel finding we report here is that the absence of the MUTYH protein confers a surprising resistance to killing by combined 6-TG/UVA exposures. *Mutyh^−/−^* MEFs are resistant to this treatment despite accumulating more DNA 8-oxoG than their wild-type counterparts. This observation clearly excludes 8-oxoG as a contributor to 6-TG/UVA toxicity. Since expression of either wild-type or inactive MUTYH proteins had similar sensitizing effects, the DNA glycosylase activity of MUTYH is also irrelevant in this regard. We suggest that the presence of the MUTYH protein either promotes the formation of, or prevents the removal of toxic DNA lesions.

MUTYH acts at replication forks to initiate correction of a specific replication error [4] and it is difficult to envisage how it might promote the formation of DNA lesions. One possible route to DNA damage is *via* the formation of DNA-protein crosslinks. We note in this regard that replication fork-associated proteins, including PCNA, RPA and MSH2/MSH6, are among identified targets for DNA crosslinking by 6-TG/UVA [36]. It is possible that the interactions of MUTYH with components of the replication machinery, specifically the MutSα MMR complex, make it vulnerable to crosslinking to DNA - embedded 6-TG. The observation that the protective effects of Msh2 and Mutyh knockouts are epistatic is consistent with this possibility. It is noteworthy that the base substitutions in the MUTYH variants that sensitize the KO MEFs map outside the MSH6 binding site (aa 246-268) [28], and all these variants retain the MutSα-interacting sequences.

The effects on cell cycle are also consistent with the formation of DNA damage that is difficult for the cell to process and induces DSBs as secondary lesions. The absence of MUTYH appears not to affect the early response to 6-TG/UVA and CHK 1 activation occurs apparently normally. At later times, whereas *Mutyh*-null cells transit S phase normally, those expressing either wild-type or G396D mutant MUTYH undergo a prolonged S phase arrest, consistent with the presence of replication-blocking DNA damage. This behaviour is correlated with reduced γH2AX formation, fewer MNs and NPBs and less apoptosis in the *Mutyh-*null cells. All these observations are consistent with reduced levels of replication-associated DSBs in 6-TG/UVA-treated *Mutyh^−/−^* MEFs. MUTYH-dependent formation of toxic secondary DNA lesions would function as a potential anti-tumour barrier. We suggest that *Mutyh*-null cells, even though less chromosomally unstable, would however accumulate point mutations derived from persistence of 8-oxoG in the genome.

Analysis of DNA 8-oxoG identifies some differences between *in vitro*/*in vivo* studies. In comparison to wild-type, *Mutyh^−/−^* MEFs cultivated *in vitro* have higher steady-state levels of DNA 8-oxoG whereas this difference is not apparent in the skin and other organs of *Mutyh^−/−^* mice [37,15]. It seems that *in vitro* culture conditions (rapid proliferation rate, high oxygen tension) emphasize the protective role of Mutyh. UVA induces DNA 8-oxoG [38,39]. In agreement with this, multiple UVA treatments resulted in higher steady-state DNA 8-oxoG levels in the skin of *Mutyh* KO mice. This increase was not observed in cultured MEFs. It seems likely that this reflects the different effects of a single *vs* multiple exposures to a low UVA dose.

In other respects, the effects of *in vivo* Mutyh abrogation on Aza/UVA-induced toxicity paralleled those observed *in vitro*. UVA increased the systemic toxicity of Aza and the minimal toxicity associated with long-term exposure to Aza was significantly exacerbated by UVA radiation in wild-type mice. Skin DNA photodamage induces the release of immunosuppressive cytokines with system wide effects [40]. We have previously shown that the inflammatory response of *Mutyh^−/−^* mice may be aberrant under some conditions [41]. Whether this phenotype influences their differential sensitivity to DNA 6-TG-related photodamage remains to be ascertained. The two squamous cell carcinomas we observed arose in 6-TG/UVA treated *Mutyh^−/−^* mice. Studies in cultured cells consistently indicate that 8-oxoG is not responsible for toxicity or chromosomal instability induced by 6-TG/UVA. Exome sequencing will ultimately reveal whether 8-oxoG contributes significantly to tumor development associated with DNA 6-TG and UVA exposure. Although the limited size of the experiment (15/genotype) clearly mandates caution, we suggest that a negative aspect of the tolerance of *Mutyh^−/−^* mice to the cytotoxic effects of Aza/UVA is an increased risk of skin cancer. Notwithstanding the precise mechanism by which tumors arise, the synergistic toxicity of Aza/UVA *in vivo* might have implications for patients undergoing thiopurine immunosuppression.

## MATERIALS AND METHODS

### MEFs cultures and cell treatment

*Mutyh^−/−^* MEFs were transfected with pYMv200–MUTYH vectors containing the wild-type or mutated human *MUTYH* cDNA (MutYγ3) which express the nuclear isoform 4 as described in ref. 16. All MEFs were grown in DMEM supplemented with 10% fetal bovine serum and 1% penicillin–streptomycin (standard medium) at 37°C and 5% CO_2_.

For treatment cells were seeded at an appropriate density and then grown for 48h or 24h in standard medium supplemented with 6-TG (Sigma Chemical Co., St. Louis, MO, USA) (concentration may vary depending on type of following analysis). After medium was removed, and cells were irradiated on ice with “UV 250W Blacklight Hand Lamp” (UV Light Technology Limited, Birmingham, England) with a UV light spectral output within the wavelength range 315-405 nm (UVA). The UVA intensity used was 10 kJ/m^2^ unless otherwise indicated.

### Clonal assay

Survival was determined by clonal assays following single and combined UVA and 6-TG treatments. Cells, seeded in 60mm dishes, were grown for 48h in standard medium supplemented with 60nM 6-TG and then irradiated with UVA (5 or 10 kJ/m^2^). After ten days cells were fixed in ethanol, stained with Giemsa and clone number was evaluated.

### Determination of DNA 8-oxoG and 6-TG

8-OxoG was measured by high-performance liquid chromatography with electrochemical detection (HPLC-EC) as described in [42]. Incorporation of 6-TG into DNA was measured by HPLC and UV absorption spectrum (250-450 nm) as previously described [43].

### Western blot analysis

To quantify the MUTYH protein by western blotting, cells were lysed in 50 mM Tris–HCl (pH 7.5), 150 mM NaCl, 1% Triton X-100, 1 mM EDTA and proteins (40 μg) were loaded on NuPAGE Novex 4-12% Bis-Tris Protein Gels (Life Technologies, Thermo Fisher Scientific, Waltham, Massachusetts, USA). MUTYH signals were normalized to the β-tubulin.

For Chk1 activation cells were irradiated with UVA after a 48h growth in standard medium supplemented with 0.6μM 6-TG. For western blotting analysis, cells were collected 30 min, 1h, 3h and 6h after UVA exposure, lysed in 2x electrophoresis sample buffer by sonication and proteins were loaded on 8% SDS-PAGE gels.

For analysis of γH2AX, cells were grown for 48h in DMEM 10% FBS 300 or 60nM 6-TG and then irradiated with UVA. Samples were collected at 0, 3h, 6h and 24h after UVA exposure. For western blotting, nuclei were extracted by suspending cells in buffer A (10mM Hepes pH 7.9, 1.5 mM MgCl_2_, 10mM KCl, 10% Glycerol, 50mM NaF, 340mM Sucrose with addition just before use of protease inhibitor, 1mM DTT and TritonX-100 0,1%), nuclei were lysed in buffer B (3mM EDTA, 0.2mM EGTA, 50mM NaF with addition just before use of protease inhibitor and 1mM DTT) and then chromatin was sonicated in 1x electrophoresis sample buffer. Proteins were loaded on NuPAGE Novex 4-12% Bis-Tris Protein Gels.

Antibodies used were: Mutyh (Abcam, Cambridge, UK), Lamin B1 (Abcam), β-tubulin (Sigma Chemical Co.), Phospho-Ser345-Chk1 (Cell Signaling, Danvers, MA, USA), Chk1 (Santa Cruz Biotechnology, Dallas, Texas, USA), γH2AX (Millipore, Temecula, CA, USA).

### Cell cycle analysis

After 6TG/UVA treatment 1-3×10^6^ cells were centrifuged, washed once in sample buffer (PBS1x, glucose 1g/L), and suspended by vortexing and slowly adding 1ml of ice-cold 70% ethanol drop-by-drop to the pellet. Cells were fixed O/N at 4°C, then vortexed for few seconds and centrifuged. Pellets suspended in 1ml of sample buffer, 50μg/ml propidium iodide (PI), 10μg/ml RNAse A were incubated for 30′ at room temperature and analyzed by flow cytometry (FACScan, BD Biosciences, San Jose, CA, USA).

### Cytokinesis-blocked micronucleus assay

Cells were grown for 48h in standard medium supplemented with 15, 30 or 60nM 6-TG and then irradiated with UVA. Cytochalasin B (Sigma), 4.5 μg/ml, was added in the medium after irradiation, for 24h, then cells were collected and spun onto microscope slides using a cytocentrifuge (Thermo Scientific). Smears were air-dried, fixed 10 minutes in methanol and stained in 4% Giemsa phosphate buffer. Cells were analyzed in the comprehensive micronucleus test as in (30). The frequencies of binucleated cells with MN and NPBs were determined analyzing 500 binucleate cells with a wellpreserved cytoplasm from two slides. The nuclear division index, a cell proliferation index, was determined in 500 cells: [mononucleated cells+(binucleated cells x2)+(trinucleated cells x3)+(tetranucleated cells x4)]/500. Apoptotic cells, having 4 or more than 4 MN, were considered in the analysis.

### Mice

A colony of *Mutyh^−/−^* and littermate wild-type mice was maintained at the animal facility of Istituto Superiore di Sanità. All studies were conducted in accordance with the principles and procedures outlined in the EU (European Community Guidelines for Animal Care, DL 116/92, application of the European Communities Council Directive, 86/609/EEC), FELASA, and ARRIVE guidelines. The animals were kept under standardized temperature, humidity, and lighting conditions, and had free access to water and food. All efforts were made to reduce the number of animals used and to minimize their suffering. For treatments animals were given 15mg/kg Aza *i.p.* and 1h after injection a UVA dose of 150 kJ/m^2^ was applied on the dorsal shaved skin with “UV 250W Blacklight Hand Lamp” (UV Light Technology Limited). All procedures were repeated three times/week for 12 months. During UVA irradiation cages were cooled by an electric fan. When suffering animals were sacrificed by cervical dislocation. At the end of treatment (12 months) organs were removed and quickly frozen in dry ice for further analyses. At the end of treatment (12 months), autopsies of carcinoma-bearing mice were performed. Normal appearing skin and skin tumors were fixed in 10% of buffered formalin, dehydrated in an ethanol series, cleared in xylene, and embedded in paraffin. Five μm sections were stained with hematoxylin and eosin and histologically evaluated by a pathologist. Other organs were removed and quickly frozen in dry ice for further analyses

### Statistical analysis

Survival and 8-oxoG levels were compared by Student *t*-test. MN, NPBs and apoptotic cells with χ^2^ test and Kaplan Meier's curves with the Log-rank test.

## SUPPLEMENTARY MATERIAL, FIGURES


