# Supplementary material for: MUTYH mediates the toxicity of combined DNA 6-thioguanine and UVA radiation
Source: Oncotarget. 2014 Dec 2;6(10):7481–92. doi: 10.18632/oncotarget.3037 (PMC4480694; doi:10.18632/oncotarget.3037)
Supplement: Supplementary file 1 [file oncotarget-06-7481-s001.pdf]

MUTYH mediates the toxicity of combined DNA 6-thioguanine and UVA radiation

Supplementary Material

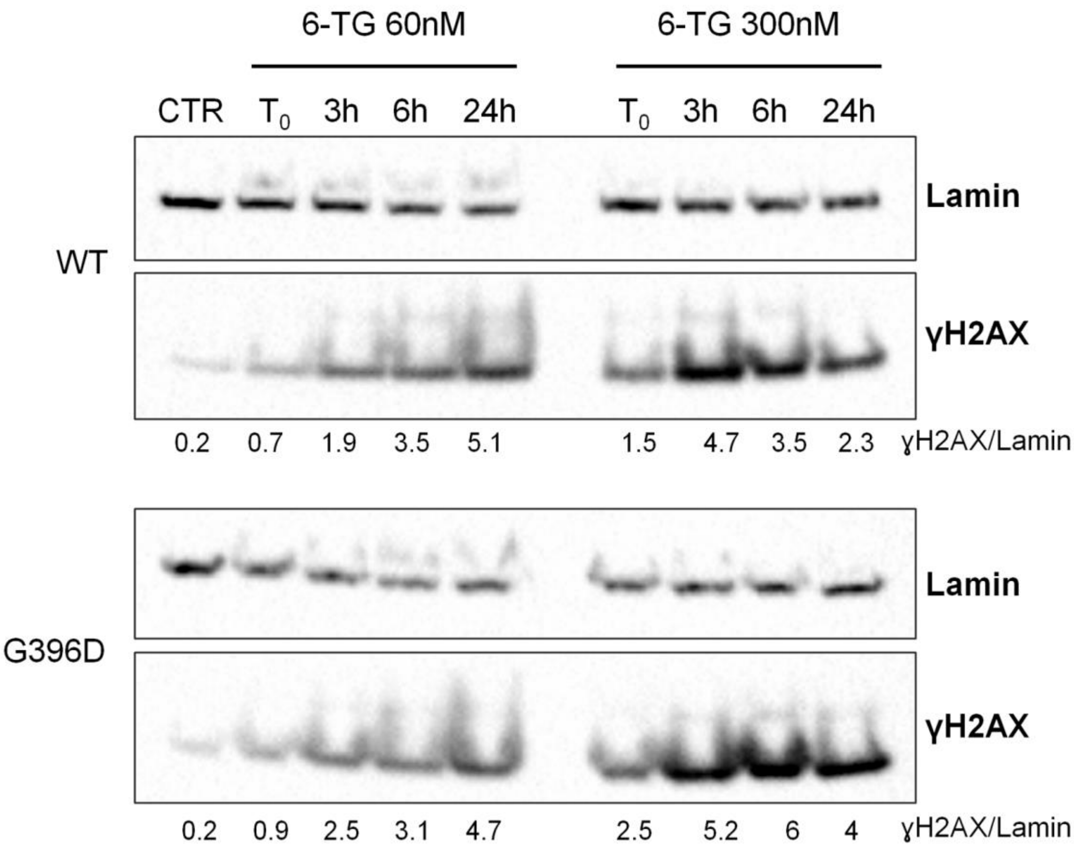

Figure S1. A representative western blotting of ɣH2AX and Lamin proteins in WT and G396D-expressing MEFs at various time points after 6-TG/UVA treatment (48h growth in 60nM or 300nM 6-TG followed by UVA irradiation). Under the blot values of normalized ɣH2AX expression as ɣH2AX/Lamin ratio are shown.
